# The histopathology of septic acute kidney injury: a systematic review

**DOI:** 10.1186/cc6823

**Published:** 2008-03-06

**Authors:** Christoph Langenberg, Sean M Bagshaw, Clive N May, Rinaldo Bellomo

**Affiliations:** 1 Department of Intensive Care, Austin Hospital, Studley Rd, Heidelberg, Melbourne, Victoria 3084, Australia; 2 Howard Florey Institute, University of Melbourne, Grattan St, Parkville, Melbourne, Victoria, Australia; 3 Division of Critical Care Medicine, University of Alberta Hospital, University of Alberta, 112 Street NW, Edmonton, Alberta, BBT6G2B7, Canada; 4 Department of Medicine, Melbourne University, Grattan St. Parkville, Victoria 3052, Melbourne, Australia

## Abstract

**Introduction:**

Sepsis is the most common trigger of acute kidney injury (AKI) in critically ill patients; understanding the structural changes associated with its occurrence is therefore important. Accordingly, we systematically reviewed the literature to assess current knowledge on the histopathology of septic AKI.

**Methods:**

A systematic review of the MEDLINE, EMBASE and CINHAL databases and bibliographies of the retrieved articles was performed for all studies describing kidney histopathology in septic AKI.

**Results:**

We found six studies reporting the histopathology of septic AKI for a total of only 184 patients. Among these patients, only 26 (22%) had features suggestive of acute tubular necrosis (ATN). We found four primate studies. In these, seven out of 19 (37%) cases showed features of ATN. We also found 13 rodent studies of septic AKI. In total, 23% showed evidence of ATN. In two additional studies performed in a dog model and a sheep model there was no evidence of ATN on histopathologic examination. Overall, when ATN was absent, studies reported a wide variety of kidney morphologic changes in septic AKI – ranging from normal (in most cases) to marked cortical tubular necrosis.

**Conclusion:**

There are no consistent renal histopathological changes in human or experimental septic AKI. The majority of studies reported normal histology or only mild, nonspecific changes. ATN was relatively uncommon.

## Introduction

Acute kidney injury (AKI) is a common clinical problem in critically ill patients [1,2]. Sepsis is the most important contributing factor for the development of AKI in the critically ill population [3]. Little is known about the pathogenesis of septic AKI. Renal hypoperfusion, and ischemia followed by acute tubular necrosis (ATN), have been repeatedly proposed as central to septic AKI development [4,5]. Recent studies, however, have shown that this paradigm might not be correct in all circumstances [6,7].

A possible strategy aimed at gaining better insight into the pathogenesis of septic AKI could be based on developing a clearer appreciation of the histopathological changes that occur in this condition. For example, if ATN was a consistent histopathological finding, this would strongly suggest that ischemia and tubular cell necrosis are probably an important pathogenetic mechanism. Regrettably, however, no comprehensive review of the histopathological features of septic AKI has yet been performed.

Accordingly, we systematically evaluated all available human and experimental studies describing kidney histopathology in septic AKI.

## Methods

Two individuals (CL and SMB) independently identified published articles on the histopathology of septic AKI using both electronic and manual search strategies. An initial screen of identified abstracts was performed followed by a full-text screen of each article identified. Our search was supplemented by scanning the bibliographies of all recovered articles.

The databases MEDLINE (1966 to December 2006), EMBASE (1980 to December 2006) and CINAHL (1982 to December 2006, week 2) were searched. PubMed was also searched. This comprehensive search was updated in July 2007. Only articles written in English were considered.

Three comprehensive search themes were derived. The first search theme was performed using the term 'OR' with the following medical subject headings and text words: 'acute renal failure', 'acute kidney failure', 'acute tubular necrosis', 'kidney dysfunction'. The second search theme was carried out using the term 'OR' with the following medical subject headings and text words: 'sepsis', 'septicemia', 'septic shock', 'bacteremia', 'lipopolysaccharide', 'cecal puncture ligation', 'endotoxin', and 'gram negative'. The final search theme was performed using the term 'OR' with the following medical subject headings and text words: 'pathology', 'histology', 'histopathology', 'microscopy', 'morphology', 'biopsy', 'cytopathology', and 'tubular necrosis'. These three search themes were then combined using the Boolean operator 'AND'.

### Study selection

Two individuals independently evaluated all identified articles for eligibility on the basis of four criteria: articles that reported original data from a primary publication, articles that reported on human subjects or experimental models, articles that made specific mention of histopathology in AKI, and articles that included subjects or models with sepsis. Any disagreements on article inclusion were resolved by discussion.

### Data extraction and synthesis

The data extracted included the number of patients or animals, the proportion with sepsis, the proportion with AKI, details of sepsis (underlying disease), details of models of sepsis in animals, biopsy/postmortem, the method of assessing samples, histology results, and mortality outcome.

Experimental models were classified as having either ATN or no ATN based on the described histopathology. We used the definitions described by Thadhani and colleagues [8]. Binary data were statistically compared using Fisher's exact test with *P *< 0.05.

## Results

**Figure 1:**
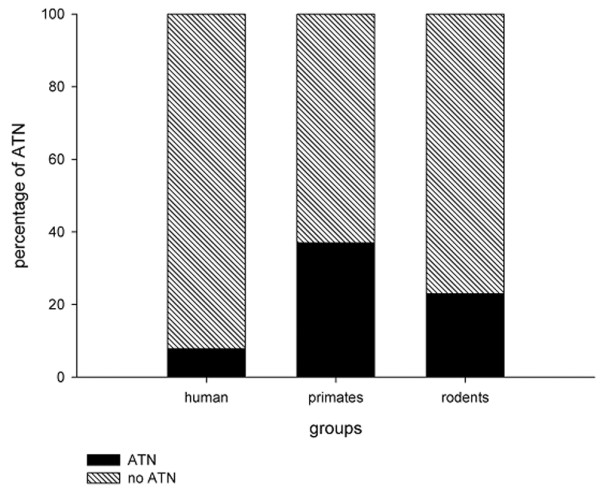
Histogram presenting the percentage of specimens showing ATN from different groups of mammals. Humans appear lest likely to have ATN. ATN, acute tubular necrosis.

Our initial search strategy yielded 378 papers. Only 73 articles, however, were identified as potentially relevant and were reviewed further. In total, 20 papers were included in our study (Figure 1). Of these, six papers were human studies while 14 studies were performed in animals.

We found six human studies examining the renal histopathology of septic AKI (Table 1). These studies were heterogeneous in design, in their definitions for AKI and in their histopathologic findings. While sepsis was attributed as the principal precipitant of AKI in all studies, there was potential for several additional confounding factors. For example, Mustonen and colleagues included only patients with systemic infection, hypovolemia or shock [9], whereas Hotchkiss and colleagues included only patients with septic AKI who were already dead [10]. In the retrospective analysis by Diaz de Leon and colleagues, renal biopsies were performed 6 to 7 days after AKI onset in 40 septic patients (37%, *n* = 107 with AKI) [11]. The results are therefore potentially biased due to late sampling and confounding from cointerventions (that is, high-dose furosemide). Finally, three studies regrettably only included a small number of septic patients [12-14].

**Table 1:** Human studies

Study	Cause	Acute kidney injury definition	Method	Cases of AKI/number of patients (%)	Acute tubular necrosis (%)
Hotchkiss and colleagues [10]	Sepsis/septic shock	Serum creatinine >2 mg/dl and urine output <20 ml/kg/hour × 6 hours	Postmortem	12/20 (60)	1 (5)
Sato and colleagues [13]	Sepsis	Not available	Postmortem	6/6 (100)	1 (17)
Mustonen and colleagues [9]	Sepsis/shock/hypovolemia	Not available	Biopsy	57/57 (100)	4 (7)
Rosenberg and colleagues [12]	Sepsis	Serum creatinine >3.5 mg/dl and urine/plasma osmolality >1	Biopsy	1/1 (100)	0 (0)
Zappacosta and Ashby [14]	Sepsis	Not available	Biopsy	1/1 (100)	0 (0)
Diaz de Leon and colleagues [11]	Severe sepsis	Serum creatinine, urine output, urine/plasma osmolality (not specified)	Biopsy	107/332 (32)	20 (50)^a^

^a^Renal biopsy was only performed in 40 patients (37% of the acute kidney injury (AKI) cohort, 12% of the total cohort).

In addition, there were two different methods for acquiring kidney histology specimens across these studies: primary renal biopsy or postmortem examinations.

Overall, of the 417 septic patients included in these six studies, only 44% (*n* = 184) had evidence of AKI; however, variable definitions were used across studies. Of these 184 patients, only 64% (*n* = 117) had histopathologic specimens available for evaluation. In total, 26 (22%) patients had features of classic ATN (Table 1). In three studies, renal biopsies were taken to assess histology. Three studies obtained renal histology by means of postmortem examinations following a standardised protocol. The remaining two studies performed standardised postmortem examinations. In studies with postmortem examination, only 11% (*n* = 2/18) of patients had evidence of ATN; whereas in studies where biopsy was performed, 24% (*n* = 24/99, *P *= 0.43) of patients showed evidence of ATN.

Mustonen and colleagues showed that nonspecific tubulointerstitial renal changes were the predominant histopathologic finding [9]. In total, 82% of specimens showed acute tubulointerstitial nephropathy, whereas 7% showed acute glomerulonephritis, 3.5% showed acute pyelonephritis and only four (7%) cases showed classic histopathologic findings consistent with ATN [9]. Similarly, Diaz de Leon and colleagues showed that 11 (27.5%) patients had nonspecific tubular or glomerular damage, whereas nine (22.5%) cases had evidence of vascular involvement [11].

In the postmortem study by Hotchkiss and colleagues, only one patient with septic AKI (8.3%) showed evidence of ATN [10]. In the study by Sato and colleagues, five out of six patients showed evidence of mild nonspecific general cell injury and only one patient had evidence of ATN [13]. Rosenberg and colleagues found mild nonspecific renal changes but no features consistent with ATN [12].

### Primate models

We found four studies describing the histopathology of septic AKI in primate models of sepsis (Table 2). In two studies (*n* = 12), there was evidence of nonspecific tubular damage in 11 animals (92%). Only one specimen revealed ATN [15,16]. In another study, after 48 hours of sepsis, renal histopathology showed evidence of edematous tubular epithelium with the tubules filled with amorphous material; however, no animal had evidence of ATN [17]. Finally, in the study by Welty-Wolf and colleagues all animals (*n* = 6) showed features suggestive of ATN [18].

**Table 2:** Primate studies

Study	Cause	Cases of acute kidney injury/number of animals (%)	Acute tubular necrosis (%)
Carraway and colleagues [17]	Heat-shocked *Escherichia coli *and live *E. coli*	6/6 (100)	0 (0)
Coalson and colleagues [16]	*E. coli *endotoxin infusion	4/4 (100)	1 (25)
Coalson and colleagues [15]	Live *E. coli *infusion	3/8 (38)	0 (0)
Welty-Wolf and colleagues [18]	Heat-shocked *E. coli *and live *E. coli*/gentamicin administration	6/6 (100)	6 (100)

Overall, in experimental primate models of septic AKI, only 37% (*n* = 7/19) of animals available for analysis showed evidence consistent with of ATN.

### Rodent models

We were able to identify only 13 relevant experimental studies in rats (Table 3). The majority did not describe the histopathology of individual specimens in detail. We therefore classified animals into those with ATN and those without ATN. Only three studies (23%) described evidence of ATN. The remaining 10 studies described a variety of histopathologic changes that ranged from normal histology to generalised renal inflammation.

**Table 3:** Rodent studies

Study	Induction of sepsis	Acute tubular necrosis
Hurley and colleagues [32]	Salmonella enteritidis endotoxin	No
Miyaji and colleagues [33]	Cecal ligation perforation/lipopolysaccharide	Yes
Sato and colleagues [34]	*Escherichia coli*	No
Hayashi and colleagues [35]	Lipopolysaccharide-induced sepsis	No
Tsao and colleagues [36]	Lipopolysaccharide-induced sepsis	No
Kadkhodaee and Qasemi [37]	Lipopolysaccharide-induced sepsis	Yes
Zager and Prior [38]	*E. coli *septicemia	No
Wang and colleagues [39]	Lipopolysaccharide-induced sepsis	Yes
Tiwari and colleagues [40]	Lipopolysaccharide-induced sepsis	No
Gallos and colleagues [41]	Cecal ligation perforation	No
Kikeri and colleagues [42]	Lipopolysaccharide-induced sepsis	No
Yokota and colleagues [43]	Lipopolysaccharide-induced sepsis	No

### Remaining animal studies

We identified two additional experimental studies, one performed in a dog model and one in a sheep model of septic AKI. In a sheep model of cecal-ligation perforation-induced sepsis, Linton and colleagues found no consistent changes in tubular cells and no evidence of ATN [19]. Hinshaw and colleagues used a dog model of septic AKI and broadly described generalised vascular congestion, often accompanied by hemorrhage, in renal tissue, but found no evidence of ATN [20].

## Discussion

We performed a systematic review of the literature using comprehensive search terms to evaluate all human and experimental studies of septic AKI describing renal histopathology. Our principal objective was to determine the nature of the typical histopathological changes seen in septic AKI. In particular, we wanted to evaluate the prevalence of features suggestive of ATN, a widely accepted marker of renal ischemia, in septic AKI to determine whether this was a potential clue to the mechanisms responsible for cell injury in septic AKI. We found very few human or experimental studies, however, which focused on the renal histopathology of septic AKI. We also found that these studies failed to show a consistent or typical renal histopathological pattern. Finally, while the majority of studies showed some general but mild histopathological changes, ATN was relatively uncommon in these human studies and only slightly more common in these experimental investigations. We believe these observations have important clinical and research implications.

The most striking finding of our study is that histopathologic data were evaluated from only 117 patients in total. Considering that an estimated 5% of all intensive care unit patients have severe AKI and that approximately 50% of AKI is primarily due to sepsis [1], one could estimate that more than 100,000 patients will have septic AKI every year in developed countries. Clearly the study sample (117 specimens overall) is inadequate to make robust inferences about the population of intensive care unit patients with septic AKI. The absence of more human data describing the renal histopathology of septic AKI is most probably related to concern about the risk of renal biopsy in acutely ill patients and to the lack of specific treatment options.

Despite such limited human data on the histopathologic changes associated with septic AKI, our review of the available evidence would suggest that ATN might be uncommon in this setting. Indeed, the most striking observation is that there is much heterogeneity of histopathological findings, ranging from totally normal to severe ATN. These observations are consistent with the heterogeneity of sepsis as a clinical condition, and they suggest caution in attributing a particular type of structural injury to this syndrome. Our findings – by failing to confirm the widely held assumption that ATN is the most common or typical histopathological substrate of septic AKI – also challenge the view that ischemia and consequent cell necrosis are most responsible for the loss of the glomerular filtration rate [4]. In fact, only 22% of human renal histopathologic specimens identified in our review showed evidence of ATN. Moreover, in the two studies evaluating postmortem specimens, where one might expect a more significant degree of ATN, only 11% showed evidence of ATN – compared with the 24% found in biopsy specimens. Nonetheless, the paucity of data on the histology of septic AKI in humans naturally led us to evaluate the renal histopathologic findings seen in experimental models of septic AKI.

In primate experimental models of septic AKI, 37% showed evidence of ATN. In particular, Welty-Wolf and colleagues showed a higher incidence of ATN. They present the only study to use vasoactive drugs to maintain blood pressure [18]. Cardiac output was not measured, and therefore we cannot be certain of whether there was a concomitant cardiogenic component to renal injury (hypodynamic sepsis). In addition, the administration of aminoglycosides may further confound the association [21]. Nonetheless, similar to primate studies, 23% of studies performed in rodents showed features consistent with ATN. Again, this would appear to be a considerably higher rate than described in the human data. There are, however, plausible explanations for these differences. Specifically, the methods for sepsis induction, the duration of sepsis prior to tissue sampling and the general supportive conditions of the experimental models (that is, systemic hemodynamics, fluid resuscitation) may contribute to significant heterogeneity across studies.

Unfortunately, in most of the studies, there were limited data provided on systemic hemodynamics such as cardiac output. Several of these models may therefore have been characterised by hypodynamic shock with decreased cardiac output, which would combine the effect of cardiogenic shock with septic shock. This hemodynamic pattern is not representative of the classical hemodynamic pattern found in human septic shock, where the circulation is generally hyperdynamic, characterised by an *elevated *cardiac output [22-28]. Recent evidence suggests that cardiac output may be the most important determinant of renal perfusion and that a hypodynamic circulation is likely to be a significant confounder in experimental models of septic AKI [6,7]. In contrast, experimental models of hyperdynamic sepsis (preserved or elevated cardiac output) have shown significant increases in global renal blood flow, decreases in renal vascular resistance and maintenance of renal ATP levels [29-31].

The present review has strengths and limitations. To our knowledge, this is the first study to comprehensively appraise the available English literature on the renal histopathologic changes associated with septic AKI. While our study is strengthened by performing a systematic and reproducible search and by using predefined study inclusion criteria, we only evaluated studies published in the English language. We recognise this may have contributed to omission of additional small investigations reported in other languages.

In addition, we used criteria for describing classic ATN as proposed by Thadhani and colleagues [8]; we recognise that if we used a broader definition for ATN, by incorporating more subtle renal histopathologic changes (that is, endothelial injury, evidence of apoptosis), the sensitivity of our search would probably have been increased. Our study was primarily focused, however, on describing the occurrence of classic ATN in septic AKI.

Finally, we acknowledge that many of the studies included (both experimental and human) were observational, were small, were limited in design (that is, no controls), were published several decades ago, and showed findings with considerable heterogeneity. Therefore, while these studies may present a biased perspective and global inferences may be limited, we cautiously question the strength of association of evidence of classic ATN in septic AKI and draw attention to the urgent need for a broader understanding to the renal histopathologic correlation in septic AKI.

## Conclusion

The available experimental and human evidence does not, at present, support the notion that ATN is the typical histopathological lesion associated with septic AKI. Experimental findings further support the notion that ATN might be relatively uncommon in sepsis. Moreover, the reviewed studies also suggest no specific or characteristic histological features exist that are reliably associated with septic AKI. In fact, if a typical histopathological pattern exists, it is one of great heterogeneity.

A complete understanding of the histopathology of any disorder represents a fundamental step in comprehending its pathogenesis and is needed long before the development of potential therapeutic interventions. Evidence of histopathologic correlation between ATN and septic AKI, from the data available, would appear weak and lacking in robustness. We contend that further investigations of validated experimental models of septic AKI along with autopsies studies in human septic shock are clearly needed to better evaluate the true renal histopathologic appearance (along with temporal trends) associated with septic AKI.

## Key messages

• Only a very small number of renal biopsies or renal postmortem assessments have been reported in humans with septic AKI.

• In these human studies, ATN was a relatively uncommon (<25%) finding.

• A limited number of experimental studies have reported the histopathological findings of septic AKI.

• In experimental studies, ATN was also a relatively uncommon histopathological finding.

• Across these experimental and human studies, there appears to be no single typical renal histopathological finding associated with septic AKI. The heterogeneity of histopathology in this condition (from normal to severe ATN) is striking.

## Abbreviations

AKI = acute kidney injury; ATN = acute tubular necrosis.

## Competing interests

The authors declare that they have no competing interests.

## Authors' contributions

CL and SMB designed the study protocol, performed the literature search, evaluated studies, extracted data, analysed data and wrote the manuscript. RB and CNM aided in the study design, and provided critical review of successive drafts of the manuscript.
